# The Hospital Journals and Gazettes

**Published:** 1919-11-08

**Authors:** 


### THE HOSPITAL JOURNALS AND GAZETTES.

### Guy's.

The London medical student of one hundred years ago continues his diary, and refers to the Bill then going through the House to regulate the practice of surgeons. "Its main object is to prevent any persons practising in future as surgeons, except members of the college; of course not affecting those already in practice. I understand that it also lays a very considerable stamp duty on the Indentures of Apprentices, compels a longer attendance on the practice of a hospital than is now required previous to an examination for a diploma, and some other clauses calculated to increase the respectability and, after a time, the practice of those who are already in the profession, by preventing the supply from exceeding the demand, as it has hitherto done." The writer says that he has seen many an attentive pupil change in one night into "a lazy member of the Royal College of Surgeons."

### St. Bartholomew's.

Mr. McAdam Eccles contributes a paper on "The Cinematograph in Medical Education and Research," and predicts that the epidiascope and the 'cinematograph are long will be side by side in every up-to-date theatre of a medical school, and the lecturer will pass rapidly from the one to the other for illustration of his theme. The relationship of the film with radiology is in its infancy, but the field here is large. The tracing of the electro-cardiograph shown with the cinematograph reproduction of the actual simultaneous movements of the heart will be an advance of considerable scientific value.

On the lighter side of the journal there is an amusing description of the trials of a temporary acting junior H.P., which contains several good things. A girl from the surgical side, with her elbow in plaster, is having her chest examined. Plaster is noticed to be cracked. "I didn't know it was cracked, doctor," she explains. "It was all right last fortnight, when I changed my vest!"

### St. Mary's.

"One of the most striking signs of the times is the plethora of doctors for any vacant appointment that carries with it a fixed salary. There is no evidence anywhere of the anticipated shortage. It may be that the public, which is not too grievously afflicted, has learnt to do without us, or it may be that the tendency evident before the war to escape from general practice to a salaried post has received an impetus from men's experience during the war. . . . In the Asylums Service the terms offered are practically the same as before the war, and take no account of the increased cost of living, and yet there are two men for every vacancy, while the competition for public-health appointments and the like is extremely keen."

### The London.

"Talking of the wards, what a fearful thing is the prevalence of the tea-drinking habit among nurses! It forms one of the earliest and most abiding impressions in the mind of the new (male) ward-student. At about 2 P.M. he enters the ward thirsting for the history-sheet, and is immediately conscious of the unfriendly regard of several pairs of eyes over as many raised tea-cups. He becomes a bright pink and retires until 4 P.M., when the same thing occurs again. It is rumoured that in addition to sundry little tots at other times, most nurses carry a thermo-flask, from which they refresh themselves at irregular intervals and in secret. Horrid thought, isn't it?"

We quote one of an amusing set of eleven verses after Lewis Carroll, entitled "A Base Hospital, B.E.F.":

"O Captain!" said the D.M.S.,
"Do you your letters know?
Can you extract a grant of leave
From every G.R.O.?
Or find a D.A.D.M.S.
Without a D.S.O.?"

The cartoon, which is now a feature of the *Gazette*, is of "Isaac."

